# Clinical features and lipid metabolism genes as potential biomarkers in advanced lung cancer

**DOI:** 10.1186/s12885-023-10509-x

**Published:** 2023-01-09

**Authors:** María Merino Salvador, Lara Paula Fernández, Juan Moreno-Rubio, Gonzalo Colmenarejo, Enrique Casado, Ana Ramírez de Molina, María Sereno

**Affiliations:** 1 Medical Oncology Department, Infanta Sofía University Hospital. Infanta Sofia University Hospital and Henares University Hospital Foundation for Biomedical Research and Innovation (FIIB HUIS HHEN), Paseo de Europa 34, San Sebastián de los Reyes, CP 28702 Madrid, Spain; 2 Molecular Oncology Group, IMDEA Food Institute, CEI UAM + CSIC, Madrid, Spain; 3 Precision Oncology Laboratory (POL), Infanta Sofía University Hospital, San Sebastian de los Reyes, Madrid, Spain; 4 Biostatistics and Bioinformatics Unit, IMDEA Food Institute, CEI UAM + CSIC, Madrid, Spain

**Keywords:** Lipid metabolism, Lung cancer, Advanced stages, Overall survival

## Abstract

**Background:**

Lung cancer is one of the most lethal tumors with a poor survival rate even in those patients receiving new therapies. Metabolism is considered one of the hallmarks in carcinogenesis and lipid metabolism is emerging as a significant contributor to tumor metabolic reprogramming. We previously described a profile of some lipid metabolism related genes with potential prognostic value in advanced lung cancer.

**Aim:**

To analyze clinical and pathological characteristics related to a specific metabolic lipid genomic signature from patients with advanced lung cancer and to define differential outcome.

**Methods:**

Ninety samples from NSCLC (non-small cell lung cancer) and 61 from SCLC (small cell lung cancer) patients were obtained. We performed a survival analysis based on lipid metabolic genes expression and clinical characteristics. The primary end point of the study was the correlation between gene expression, clinical characteristics and survival.

**Results:**

Clinical variables associated with overall survival (OS) in NSCLC patients were clinical stage, adenocarcinoma histology, Eastern Cooperative Oncology Group (ECOG), number and site of metastasis, plasma albumin levels and first-line treatment with platinum. As for SCLC patients, clinical variables that impacted OS were ECOG, number of metastasis locations, second-line treatment administration and Diabetes Mellitus (DM). None of them was associated with gene expression, indicating that alterations in lipid metabolism are independent molecular variables providing complementary information of lung cancer patient outcome.

**Conclusions:**

Specific clinical features as well as the expression of lipid metabolism-related genes might be potential biomarkers with differential outcomes.

## Introduction

Lung cancer is the leading cause of cancer-related death worldwide. It is also the main cause of cancer death among men and the second among women [1]. Almost 90% of cases are due to tobacco exposition but the incidence in non-smokers is increasing [2]. Some other environmental agents such as radon, asbestos, ionizing radiation and some diseases such as pulmonary fibrosis and HIV (Human Immunodeficiency Virus) have been described as possible risk factors for lung cancer [3]. Over the last few years the influence of diet is gaining importance as a risk factor for cancer, but data regarding lung cancer is highly variable. Several prior studies suggest the protective effect of fruits and vegetables while others point to an increased risk of lung cancer in patients taking beta carotene supplements [4].

About 80–85% lung cancer cases are non-small cell lung cancer (NSCLC), with the most representative subtypes being adenocarcinoma and squamous cell tumors. Small cell lung cancer (SCLC) cases account for 10–15% of all lung malignant neoplasms.

Although some relevant studies suggest lung cancer mortality reduction by performing periodical computerized tomography [5, 6], at present this screening is not incorporated into clinical practice. Therefore, the main preventive strategy is smoking cessation, especially in groups with increasing incidence such as youth and women [5, 7]. Most new lung cancer cases are diagnosed in advanced stages and the outcome is poor.

Survival is influenced by clinical, pathologic and molecular factors. Clinical stage is the most important prognostic factor with better outcome for earlier stages. Performance status at diagnosis, determined by ECOG scale, and loss of weight are also relevant prognostic factors [8]. Chemotherapy, immunotherapy and biological treatments have also a growing impact in modifying natural history of lung cancer, mainly, NSCLC.

Some genetic alterations such as activating mutations in the gene encoding EGFR (epidermal growth factor receptor), MET (mesenchymal-to-epithelial transition protein), BRAF (v-raf murine sarcoma viral oncogene homolog B1), or RET (rearranged during transfection) and ROS1 (c-ros oncogene 1) rearrangements, are response predictive factors to targeted therapy [9] improving both quality of life and survival. PD-L1 (programmed death-ligand 1) expression in tumor cells and infiltrating peritumoral lymphocytes and tumor mutation burden (TMB) have been proposed as predictive markers of response to immune check-point inhibitors. Both targeted therapy and immune check-point control inhibitors have drastically changed lung cancer treatment landscape in the last decade.

**Fig. 1:**
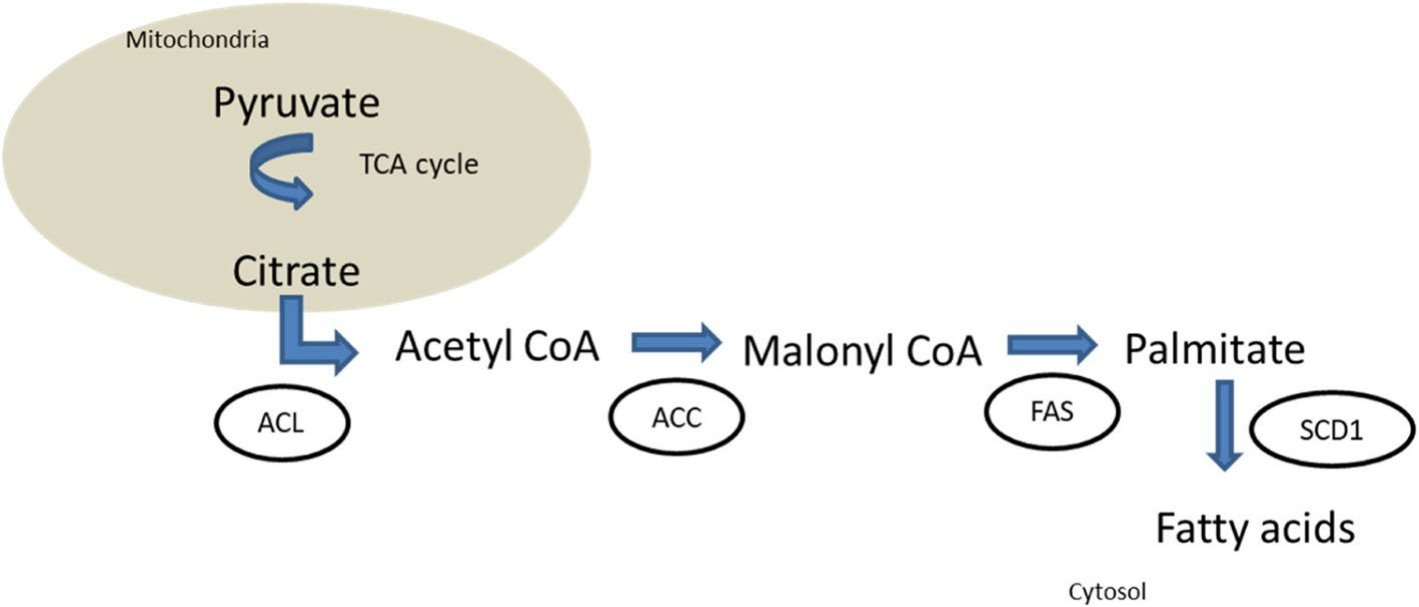
De novo lipogenesis in adipose tissues. TCA: tricarboxylic cycle. ACL: ATP-citrate lyase. ACC: acetyl-coA carboxylase. FAS: fatty acid synthase. SCD1: stearoyl-CoA desaturase-1

Tumor cells can adapt their ability to metabolize nutrients to obtain energy even in a low-energy situation. Although most of knowledge in this area concerns carbohydrate metabolism, lipid metabolism is becoming increasingly important. Cancer cells might increase de novo lipogenesis (Fig. 1). Some enzymes involved in this process such as Acetyl-CoA thioesterase (ACOT) [10] may be over expressed in cases of poor prognosis lung cancer. Fatty acid synthase (FAS) also plays a major role in adipogenesis and some FAS-expressing tumors have poorer outcomes due to higher recurrence rates [11].

Adipocytes metabolism becomes quicker when tumor cells are present and secrete metabolic substrates such as fatty acids and glycerol. These will in turn be used by tumor cells to ensure growth and survival [12].

Most studies suggest a relationship between high plasma cholesterol levels and lung cancer incidence [13]. In addition, there is growing evidence about the association between obesity and risk of different types of cancer such as breast postmenopausal tumors, colorectal, endometrial, pancreatic, biliary tract cancer, etc.…However, data on lung cancer is not conclusive, and there are even some reports that suggest an inverse association between BMI (body mass index) and lung cancer risk [14]. On the other hand, obesity is associated with an immunosuppressive phenotype and proinflammatory status that could lead to better response to immune check-point inhibitors treatment in lung cancer patients [15].

We previously described a signature of a lipid metabolism-related genes panel with prognostic value biomarkers in NSCLC and SCLC [16, 17]. In this work, we enrich previous molecular data with an analysis of clinical features, histologic characteristics and relevant laboratory parameters, as well as clinical outcomes of patients with advanced lung cancer.

## Materials and methods

### Patient´s selection

A total of 151 formalin-fixed, paraffin-embedded (FEPE) samples were obtained from a sample of NSCLC and SCLC patients from the Medical Oncology Department of Infanta Sofía University Hospital (San Sebastián de los Reyes, Madrid). Patients were enrolled from November 1, 2008 to September 31, 2017 and all cases were stage IIIB (no candidates to radical radiation) or IV. They were treated according to clinical international guidelines. Clinical, analytical and pathological data was collected from electronic medical reports. FFPE samples were studied by an anatomic pathologist confirming > 80% of tumor cells. The study was approved by the IMDEA Food Research Ethics Committee. Overall survival (OS) was defined from the date of diagnosis to the date of patient death and progression-free survival (PFS) was defined from the data of start of chemotherapy to the date of progression.

Clinical variables included were age, gender, clinical stage, histology (adenocarcinoma, squamous cell and small cell or “oat cell”), EGFR/ALK (Anaplastic lymphoma kinase) mutation status, ECOG performance status (0–1 *vs* 2–4), loss of weight and BMI. Laboratory parameters involved in cholesterol and glucose metabolism such as albumin, total, HDL (high-density lipoprotein) and LDL (low-density lipoprotein)-cholesterol levels were included. Relevant comorbidities were also analyzed, mainly, those related to metabolism like diabetes mellitus (DM) and dyslipidemia (DL). We also described tumor burden, metastasis location as well as number of metastatic sites and chemotherapy regimen. It should be noted that all patients included were treated before 2017, (pre-immunotherapy era) therefore first line treatments were based on platinum agents instead of chemo-immunotherapy combination or immunotherapy.

### Gene selection

We designed a Taq-Man Low-Density Array formed of 22 lipid metabolism-related genes as previously described [16]. Gene expression assay was performed in HT-7900 Fast-Real-time PCR. These genes were selected due to their role as regulators of cell metabolism, major steps of interconnection among lipid pathways and their reported role as potential prognostic biomarkers in several types of cancer [18].

### Statistical analysis

The primary endpoint of the study was the relationship between gene expression and survival: OS and PFS. We also assessed the potential association between clinical and histologic patient features and outcomes. The Kaplan–Meier method was used to estimate OS curves, and Cox regression analysis was used to test the association between OS, PFS and clinical variables. Absolute values and percentages were used to describe qualitative variables while mean and median were used for quantitative variables. Range and standard deviation were used as dispersion measures. Overall survival and progression-free survival has been represented using the Kaplan–Meier method and univariate and multivariate Cox regression analysis were used to assess the association between overall survival and clinical variables. Bootstrapping with 1000 samples was used to calculate an optimism-corrected C-statistic. Statistical significance was defined as *p*-value < 0.05. The Bonferroni method was applied for multiple test correction of the *p*-values.

## Results

Clinical characteristics of 151 lung cancer patients included are summarized in Table 1.

**Table 1:** Clinical characteristics of patients

Variable	Patients (%)
Median age	64
Gender	
Male	114 (75.5%)
Female	37 (24.5%)
ECOG	
0	25 (16.55%)
1	90 (59.6%)
2	33 (21.85%)
3	7 (4.6%)
4	6 (3.9%)
Diabetes Mellitus	30 (19.8%)
Metformin	16 (53%)
Dyslipidemia	47 (31%)
Statins	47 (100%)
Median BMI (kg/m2)	26,28
Total cholesterol (mg/dL)	
< 200	68 (53.54%)
> 200	12 (9.45%)
HDL-cholesterol (mg/dL)	
< 35	12 (9.45%)
> 35	57 (44.88%)
LDL-cholesterol (mg/dL)	
< 130	59 (46.45%)
> 130	10 (7.87%)
Loss of weight	30 (19.86%)
Histology	
Non-small cell	90 (60%)
Squamous cell	22 (24.5%)
Adenocarcinoma	68 (75.5%)
Small cell	61 (40%)
EGFR mutations	6 (3.9%)
ALK translocation	0 (0%)
Stage	
IIIB	32 (21.2%)
IV	119 (78.8%)
Metastasis	
No	51 (33.78%)
Yes	100 (66.22%)
Skin	8 (5.29%)
Adrenal glands	19 (12.58%)
Liver	46 (30.46%)
Bones	45 (28.8%)
Pleura	19 (12.58%)
Central nervous system	17 (11.25%)

(*ECOG* Eastern cooperative oncology group, *BMI* Body mass index, *HDL* High-density lipoprotein, *LDL* Low-density lipoprotein, *EGFR* Epidermal growth factor receptor, *ALK* Anaplastic lymphoma kinase)

We present all collected data split according to lung cancer subtype: NSCLC and SCLC.

### Non-small cell lung cancer

Ninety patients with NSCLC were included. Median age was 64 years. The majority were males (75%) and more than a half smokers (52%) or former smokers (34%). Half of patients (51%) had ECOG performance status of 1 and 18%, 24%, 3.3% and 3.7% had ECOG of 0, 2, 3 and 4 respectively. Around 70% had stage IV NSCLC and bones were the most common site of metastasis.

Median BMI was 25. Regarding comorbidities, 19% of all patients had DM and 27% DL, all of them were on statins treatment.

Less than 4% of patients had a driver mutation, thus platinum-based chemotherapy was used in first line in almost all NSCLC patients; being carboplatin/docetaxel and carboplatin/paclitaxel/bevacizumab the most widely used regimens (Table 2).

**Table 2:** Chemotherapy regimens used in patients with NSCLC (non-small cell lung cancer)

Variable	Number of patients (%)
Platinum combination (*n* = 90)	
Carboplatin-gemcitabine	5 (5.55%)
Cisplatin-gemcitabine	4 (4.44%)
Carboplatin-docetaxel	18 (20%)
Cisplatin-docetaxel	8 (8.88%)
Carboplatin-docetaxel-bevacizumab	6 (6.66%)
Carboplatin-paclitaxel-bevacizumab	12 (13.33%)
Cisplatin-pemetrexed	7 (7.77%)
Carboplatin-pemetrexed	6 (6.66%)
Cisplatin-vinorelbine	5 (5.55%)
No treatment	14 (15.55%)
Monotherapy	5 (5.55%)

The previous survival analysis performed in a series of 90 NSCLC patients had showed significant association with OS for three genes: Acyl-CoA synthetase long-chain family member 3 (ACSL3), Nidogen 1 (NID1) and Resistin (RETN) [16]. Results are summarized in Table 3.

**Table 3:** Gene expression and OS in NSCLC

Gene	HR (95% CI)	*p-value*	*p-value Bonferroni*
ACSL3	3.15 (1.61–619)	0.0002	0.004
NID1	2.39 (1.44–3.95)	0.0005	0.01
RETN	3.5 (1.74–7.05)	0.0001	0.002

(*ACSL3* Acyl-CoA synthetase long-chain family member 3, *NID1* Nidogen 1, *RETN* Resistin, *HR* Hazard ratio, *CI* Confidence interval)

Clinical variables statistically associated with both better PFS and OS, were clinical stage, adenocarcinoma histology, ECOG 0–1, number and site of metastasis and plasma albumin levels. Statins treatment for dyslipidemia only demonstrated a significant impact on PFS while first line treatment based on platinum combination, had a significant relationship with better OS (Table 4). None of the patients included received immunotherapy because it had not been approved in this setting at the moment of recruitment.

**Table 4:** Clinical variables and survival in NSCLC (non-small cell lung cancer)

Variable	HR(95% CI) OS	p OS	HR(95%CI) PFS	p PFS
Stage	3.27(1.86,5.76)	8.64e-06*	2.1(1.22,3.59)	0.005*
Adenocarcinoma vs squamous cell carcinoma	0.561(0.324,0.972	0.031*	0.392(0.216,0.714)	0.001*
ECOG	2.78(2.03,3.82)	1.03e-09*	1.94(1.36,2.77)	3.42e-04*
Metastasis	2.54(1.55,4.17)	1.37e-04*	1.82( 1.1,2.99)	0.018*
Number of metastatic sites	1.54(1.26,1.88)	1.15e-04*	1.48(1.17,1.88)	0.003*
Statins	0.772(0.449,1.33)	0.339	0.496(0.265,0.926)	0.019*
Metformine	1.71(0.806,3.61)	0.19	1.77(0.792,3.95)	0.193
First line platinum regimen	0.512(0.301,0.87)	0.01*	0.886(0.536,1.46)	0.633

(*HR* Hazard ratio, *CI* Confidence interval, *OS* Overall survival, *PFS* Progression-free survival, *ECOG* Eastern cooperative oncology group)

We assessed the potential relationship between expression of these genes statistically associated with OS in NSCLC and clinical features without finding any significant result. We also ran a multiple regression analysis to test for the effect of one variable while correcting for differences in other variables (Table 5).

**Table 5:** Multiple regression test

Variable	Beta (CI)	HR (CI)	*P* value
ACSL3	0.884 (0.174, 1.59)	2.42 (1.19,4.92)	0.009
NID1	0.605(0.0687,1.14)	1.83(1.07,3.13)	0.025
Clinical stage	1.12(0.469,1.77)	3.07(1.6,5.88)	3.04E-04
ECOG			
1 *vs* 0	0.602(-0.253,1.46)	1.83(0.777,4.3)	2.57E-06
2 *vs* 0	1.83(0.884,2.77)	6.22(2.42,16)	
3 *vs* 0	2.92(1.35,4.48)	18.4(3.86,88.1)	
4 *vs* 0	4.11(2.2,6.02)	61.1(9.03,414)	

(*ACSL3* Acyl-CoA synthetase long-chain family member 3, *NID1* Nidogen 1, *HR* Hazard ratio, *CI* Confidence interval, *ECOG* Eastern cooperative oncology group)

*P*-Value from the likelihood ratio test that compares this model with a similar one without genetic variables is 0.00012. Therefore, relationship between gene expression and OS is statistically significant and adds predictive capacity to our model.

### Small cell lung cancer

Data from 61 patients with SCLC were analyzed. Most of them were males (73%), with a median age of 65 years. More than sixty percent of patients (67%) were smokers and 24% former smokers. Median BMI was 20 and more than a half of patients had ECOG PS of 0–1 (80%) while the rest had ECOG 2 (13%), 3 (2,7%) and 4 (2.7%). Patients with diabetes were 19% and almost all of them were on metformin treatment, while all dyslipidemic patients (59%) were being treated with statins. Most patients included (78%) had stage IV disease and most common site of metastasis was the liver followed by central nervous system. Almost every patient received platinum-etoposide as first line treatment (Table 6). Median PFS was 3.6 months and median OS was 6 months.

**Table 6:** Chemotherapy regimens used in patients with SCLC

Variable	Number of patients (%)
SCLC first line treatment (*n* = 61)	
Cisplatin-etoposide	32 (52.45%)
Carboplatin-etoposide	28 (45.9%)
No treatment	1 (1.11%)

(*SCLC* Small cell lung cancer)

We had also tested the panel of 22 lipid metabolism genes in a set of 61 SCLC samples and found statistically significant association with OS for 2 genes: Alpha-Methylacyl-CoA Racemase (AMACR) and Perilipin 1 (PLIN1) [17]. Results are summarized in Table 7.

**Table 7:** Gene expression and OS in SCLC

Gene	HR (95%CI)	*p-value*	*p-value Bonferroni*
AMACR	0.214 (0.0965,0.474)	0.00043	0.009
PLIN1	0.363 (0.195,0.676)	0.001	0.031

(*AMACR* Alpha-Methylacyl-CoA Racemase, *PLIN1* Perilipin 1, *HR* Hazard ratio, *CI* Confidence interval, *SCLC* Small cell lung cancer)

While ECOG and number of metastatic locations had a relevant impact on OS, second line chemotherapy administration, DM and metformin treatment were statistically associated with both PFS and OS (Table 8).

**Table 8:** Clinical variables and survival in SCLC

Variable	HR(95%CI) OS	p OS	HR(95%CI) PFS	p PFS
ECOG	1.74(1.12,2.72)	0.022*	1.56(0.85,2.85)	0.154
Metastasis	1.84(0.836,4.06)	0.115	1.64(0.745,3.62)	0.205
Number of metastatic locations	1.28(0.858,1.91)	0.227	1.21(0.81, 1.8)	0.355
Diabetes Mellitus	3.34(1.29,8.66)	0.02*	3.28(1.28,8.39)	0.021*
Metformine	3.17(1.19,8.46)	0.034*	3.04(1.15,8.01)	0.039*
Second line treatment	0.202(0.0852,0.479)	9.17e-05*	0.174(0.0661,0.458)	8.26e-05*

(*SCLC* Small cell lung cancer, *HR* Hazard ratio, *CI* Confidence interval, *OS* Overall survival, *PFS* Progression-free survival, *ECOG* Eastern cooperative oncology group)

We also assessed the association between expression of genes significantly associated with OS in SCLC and clinical features without statistically significant results.

Finally, we tested the inclusion of second-line treatment (highly significant in the descriptive analysis) in our previously published model [17], which included an AMACR + PLIN1 genetic risk score as well as gender, age, stage, smoking status and metabolic health as predictor variables. In this model, p-value for second-line treatment was 0.0012 (Table 9).

**Table 9:** Multiple regression test

Variable	*P* Value	HR (CI)
GSC	0.0018	0.473 (0.298, 0.75)
Gender	0.825	0.928 (0.479, 1.8)
Age	0.295	1.02 (0.985, 1.05)
Smoking status	0.335	0.598 (0.269, 1.33)/2.47(0.999, 6.13)
Meth	0.005	2.37 (1.29, 4.35)
Second-line treatment	0.0012	0.28 5 (0.128, 0.638)

*GRS* Genetic risk score, *HR* Hazard ratio, *CI* Confidence interval, *Meth* Metabolic health

In addition, we validated both models using Bootstrap resampling, to assess whether the inclusion of second-line treatment provided additional predictive value. The optimism-corrected c-index was 0.701 for the previous model and 0.731 for the current one, indicating a slight increase in predictive power without overfitting.

## Discussion

In the present study 86% and 91% of patients with NSCLC and SCLC, respectively, were smokers or former smokers. This is consistent with data from occidental countries, where the rate of lung cancer patients with active tobacco use is 90% [19]. Nearly all patients treated with chemotherapy used a platinum based regimen, according to clinical guides [20]. Fifteen percent of NSCLC and 1% of SCLC patients did not receive any oncologic specific treatment at all due to poor ECOG at diagnosis. This difference might be explained because of a higher chemo sensitivity and heavier symptom burden in SCLC vs NSCLC. In clinical practice, we have observed a clear clinical benefit from chemotherapy in SCLC, even in those patients with an important ECOG deterioration due to cancer [21].

### Non-small cell lung cancer

Lung cancer mortality remains high despite recent major advances in oncology and precision medicine is one of the most promising strategies to improve survival. This requires firstly establishing specific prognostic groups to discriminate those patients with different outcome. Lipid metabolism has been raised as a potential target for cancer diagnosis, prognosis and treatment [22] and lipid metabolism alterations have been described as prognostic biomarkers in several types of cancer [23].

In our previous work, we had found that ACSL3, NID1 and RETN expression were associated with poor survival and as we showed in preclinical tests, patients could benefit of antitumor properties of statin administration. In addition, to validate these results, we also performed a parallel analysis based on ACSL3, NID1, and RETN expression in larger series of NSCLC samples from The Cancer Genome Atlas (TCGA) study, ACSL and RETN expression had an overall survival risk effect [16]. There is some preclinical evidence related to our data.

ACSLs are responsible for activating long-chain fatty acids and are frequently deregulated in cancer. They are mainly expressed in the endoplasmic reticulum and lipid droplets. Among the 5 mammalian ACSL families ACSL3 has been detected in several types of cancer and high expression correlates with worse prognosis in patients with melanoma and triple negative breast cancer [24] and might be involved in prostate carcinogenesis [25].

NID1 is a glycoprotein found in basement membranes. It is essential for structural integrity through connecting the collagen IV and laminin networks, increasing stability. Other authors have investigated the impact of NID1 in cancer suggesting a potential negative prognostic role but prospective data are lacking and, in the end, results are inconclusive. Alecovic et al.analyzed secretome of lung metastasis sublines of human breast cancer and found that overexpression of NID1 increased lung metastasis and reduced survival [26] while Ferrero et al.showed that secretion of NID1 from endothelial cells inhibited migration of human breast cancer cells SK-BR-3 [27].

Finally, RETN is a member of the cysteine-rich proteins family secreted from adipocytes and monocytes in humans and may play a role in modulating cancer pathogenesis. Previous studies found that expression increases along with the progression of some tumors including breast, prostate, colon, gastric and endometrial cancers [28]. It also promotes metastasis in chondrosarcoma and breast tumors [29, 30]. As mentioned in our previous work we demonstrated that RETN expression has a negative impact on OS in NSCLC patients. There are few data on this topic but almost all the results are consistent with our study. Zhao CC et al.assessed RETN expression in 70 pairs of lung adenocarcinomas and normal tissues and analyzed in vitro cell behavior, clinical characteristics of tumors and outcomes. They found that resistin expression was significantly associated with increased tumor size, clinical stage and lymph node metastasis, and negatively associated with PFS and OS [31]. Therefore, resistin could be a potential target for NSCLC.

In addition to our finding of a potential lipid metabolism related signature, some classical clinical and pathologic features were associated to OS. In the present analysis OS was associated with clinical stage, ECOG at diagnosis, histology, plasma albumin levels, distant metastasis and number of metastatic sites. These findings are consistent with literature. Clinical stage and performance status are the most important prognostic factors. Survival is higher for early stages with 2 years OS rate of 90% for patients with stage I tumors and around 10% for patients with stage IV [32] and patients with robust PS (performance status) live longer than those with ECOG 1 or superior [33].

Tumor burden could also influence the outcomes; patients with oligometastatic disease are more likely to undergo local aggressive treatments such as radiotherapy or surgical removal of metastasis, which could result in both longer disease-free interval and overall survival [34]. Regarding chemotherapy, platinum-treated patients had the longer OS in this series. In daily clinical practice these patients are usually younger, have ECOG PS 0 or 1 and adequate organ function without significant comorbidities. Thus, this scenario could have an impact on treatment effectiveness and survival.

Histology has a controverted role in NSCLC prognosis. While there are some data suggesting that patients with early stage lung adenocarcinoma live longer [35], most of studies attribute longer survival to patients with resected squamous cell carcinoma [36]. However, patients with advanced lung adenocarcinoma have better outcomes than squamous cell carcinoma, especially if a driver mutation is detected.

Regarding laboratory parameters, we found that serum albumin is a marker of nutritional status and can be affected by situations of inflammation. Pro-inflammatory cytokines have an impact on albumin synthesis and could contribute to low albumin levels in situations of chronic inflammation such as cancer. Adequate albumin levels are described as a prognostic factor in several types of malign tumors including lung cancer [37]. Consistently, in our series, normal serum albumin was associated with longer survival.

Smoking and loss of weight at diagnosis can also influence prognosis [37]. Additionally increased lipolysis in adipose tissues leads to cachexia, affecting 60% of lung cancer patients. Therefore, cachexia has also been proposed as a potential prognostic factor [38]. However, we did not find a significant association between neither of these parameters and survival, probably due to the small sample size.

In these days, there is a growing interest in learning about the role of statins in lung cancer. Although treatment with statins has not yet demonstrated an impact on survival in randomized clinical trials, a growing body of preclinical and observational research suggests that they have a potential as a therapeutic strategy in lung cancer [39]. Our study did not show statistically significant improvement in OS but patients who were treated with statins tend to have better clinical outcome.

We also conducted several preclinical tests to assess the anticancer effect of statins in human NSCLC cells and observed that simvastatin significantly modulated genes related to lipid metabolism and induced apoptosis activation [16]. Thus statins might have a role as targeted treatment in selected NSCLC patients.

Neither the expression of ACSL3, NID1 or RETN was associated with any clinical features. However, clinical stage and performance status at diagnosis are already recognized prognostic factors and, as we described in the main analysis, they had a statistically association with OS. Surprisingly, although gene expression had a significantly influence on outcome, it was not related to these features, supporting the hypothesis that the expression of these genes is an independent prognostic factor.

### Small cell lung cancer

A relevant lipid metabolism signature was also described in SCLC setting. As we previously showed, we found statistically significant association with better OS for two genes: Alpha-Methylacyl-CoA Racemase (AMACR) and Perilipin 1 (PLIN1) [17].

AMACR is an enzyme localized in both mitochondria and peroxisomes involved in peroxisome oxidation. There are previous studies that report an association of AMACR expression with better SCLC prognosis. Shilo K et al.reported a series of 72 SCLC where 51% were positive for AMACR. These patients had better survival than those with AMCR-negative tumors [40]. However, differences in AMCR expression have not been associated to outcomes in NSCLC [41].

PLIN1 is a protein that coats lipid droplets in adipocytes protecting them from the action of lipase. It is localized on the surface of intracellular lipid droplets. Phosphorylation of perilipin is essential for the mobilization of fats in adipose tissue and when it is unphosphorylated, blocks lipolysis [42]. It has been reported as a biomarker in some tumors such as breast, low-grade glioma, hepatocellular carcinoma and sarcoma, with a relevant prognostic value in breast tumors, but to date there was no data on lung cancer.

Besides genetic expression, we assessed the relationship between clinical and pathologic features and outcomes in SCLC. Although clinical stage and functional status are recognized prognostic factors, there are no conclusive data in the literature on the influence of other clinical parameters on survival.

In this study ECOG performance status at diagnosis, number of metastatic sites, second line treatment and DM had an impact on overall survival. Performance status and loss of weight are the most important patient-dependent prognostic factors in SCLC [8]. We were not able to find a relationship between loss of weight and survival but it could be easily explained by the fact that the proportion of patients with weight loss in our series was minimal and all of them had NSCLC. As stated above, patients with better functional status tend to live longer [33] and they usually receive full-dose treatments, with no delays, which could contribute to better outcomes. Patients who maintain good ECOG beyond progression are candidates for second-line treatment. In this study only 11 patients received second-line chemotherapy but it was still associated with statistically significant longer OS, reaching 30 months in some cases. Furthermore, it was a statistically significant variable in the multiple regression test, and by adding it, we were able to improve the predictive capacity of our previous model. However, sample size is limited and we should consider a positive selection of patients with fewer symptoms, better response to prior chemotherapy and better ECOG performance status. The most important prognostic factor in SCLC is clinical stage at diagnosis [43]. We were unable to show this relationship in our study, possibly because of underrepresentation of stage III tumors in our series.

Although we found a relationship between pre diagnosis DM and OS, there is no prospective data supporting it as a prognostic factor in SCLC, however there are quite observational studies that suggest a negative impact of DM on survival in NSCLC [44]. Metformin, an oral antidiabetic drug, has been proposed as an adjuvant therapeutic agent in lung cancer treatment because of its potential antiproliferative effect against tumor cells. Anyway, to date the results are controversial [45]. More studies are needed to confirm these findings.

Serum albumin levels have been described as a prognostic factor in SCLC [46, 47] but, opposite to NSCLC, in this SCLC sample we did not find a statistically significant impact on survival. This could have been influenced by the fact that no SCLC patient in this group had loss of weight at diagnosis which could have led to a selection bias for well-nourished patients.

As in NSCLC, there was not a significant association between AMACR and PLIN1 expression and clinical parameters, confirming again that they might be prognostic biomarkers for all patients with SCLC regardless of clinical phenotype.

Our results are largely consistent with previously reported data, therefore, even if it is a retrospective series with limited sample, it could be considered representative and might help to generate hypothesis to select prognostic groups that may benefit from specific therapeutic strategies.

## Conclusions

Lipid metabolism alterations are relevant in lung cancer development. ACSL3, NID1 and RETN expression could be independent negative prognostic markers in NSCLC while AMCR and PLIN1 might be related to better outcomes in SCLC. These results indicate that NSCLC and SCLC display different lipid-related metabolic profiles, which could complement the prognostic value of classical clinical characteristics. In addition, these findings could be important for developing potential novel treatment strategies based on the regulation of lipid metabolism.

## Data Availability

All data generated or analyzed during this study are included in these published articles: Paula Fernandez L, Merino M, Colmenarejo G, Moreno-Rubio J, Sanchez-Martinez R, Quijada-Freire A, et al. Metabolic enzyme ACSL3 is a prognostic biomarker and correlates with anticancer effectiveness of statins in non-small cell lung cancer. Molecular Oncology. 2020;14(12):3135–52. Fernandez LP, Merino M, Colmenarejo G, Moreno Rubio J, Gonzalez Pessolani T, Reglero G, et al. Metabolic Health Together with a Lipid Genetic Risk Score Predicts Survival of Small Cell Lung Cancer Patients. Cancers. 2021;13(5).
